# Loss of agency in apraxia

**DOI:** 10.3389/fnhum.2014.00751

**Published:** 2014-09-23

**Authors:** Mariella Pazzaglia, Giulia Galli

**Affiliations:** ^1^Department of Psychology, University of Rome ‘La Sapienza’Rome, Italy; ^2^IRCCS Fondazione Santa LuciaRome, Italy

**Keywords:** agency, apraxia, fMRI, rehabilitation, action

## Abstract

The feeling of acting voluntarily is a fundamental component of human behavior and social life and is usually accompanied by a sense of agency. However, this ability can be impaired in a number of diseases and disorders. An important example is apraxia, a disturbance traditionally defined as a disorder of voluntary skillful movements that often results from frontal-parietal brain damage. The first part of this article focuses on direct evidence of some core symptoms of apraxia, emphasizing those with connections to agency and free will. The loss of agency in apraxia is reflected in the monitoring of internally driven action, in the perception of specifically self-intended movements and in the neural intention to act. The second part presents an outline of the evidences supporting the functional and anatomical link between apraxia and agency. The available structural and functional results converge to reveal that the frontal–parietal network contributes to the sense of agency and its impairment in disorders such as apraxia. The current knowledge on the generation of motor intentions and action monitoring could potentially be applied to develop therapeutic strategies for the clinical rehabilitation of voluntary action.

## Evidence for the loss of sense of agency in apraxia

The sense of agency implies the subjective experience of elaboration, monitoring, and control of external events through one’s own motor actions, as well as the neural intention to act. Using cognitive neuroscience techniques, researchers have attempted to elucidate this interesting phenomenon (Farrer et al., 2003b; David et al., 2007; Spengler et al., 2009; Tsakiris et al., 2010; Salomon et al., 2013; Weiss et al., 2014) distinguishing, at the conceptual level, between two important aspects of agency: a retrospective component (the outcome of action—objective) and prospective signal (from the self-perception of generated actions to the intention to move—subjective) (Moore and Obhi, 2012; Chambon et al., 2013). Agency research has attracted investigators and theorists, although the mechanism appears very natural, critical voices have questioned the validity of studying agency via conventional scientific paradigms (David, 2012). An alternate approach is to investigate how brain damage may alter the awareness of being causally involved in an action (de Jong, 2011).

A prime neurological example is apraxia, a disturbance characterized by a marked impairment in performing volitional movements (de Jong, 2011; Dovern et al., 2011; Wolpe et al., 2014). In essence, apraxia encompasses a broad spectrum of higher-order *purposeful* movement disorders (Leiguarda and Marsden, 2000) that affect both sides of the body, even though neurological damage is more frequently associated with unilateral left frontal and parietal lesions (Haaland et al., 2000; Leiguarda and Marsden, 2000; Hermsdörfer et al., 2003). The traditional definition includes deficits in performing, imitating, and recognizing skilled actions known as meaningless or meaningful gestures (Rothi and Heilman, 1984; Pazzaglia et al., 2008a,b). The pathological condition is identified on the basis of an inability to execute both transitive (using an object) and intransitive (without an object) gestures with different body effectors (mouth, hand, or foot) (Leiguarda and Marsden, 2000). This failure to move intentionally cannot be explained by primary motor or sensory impairments, or by deficits in memory or comprehension (De Renzi and Lucchelli, 1988).

Apraxia has been, and is still, subject to intense debate about its deficits to sensorimotor function and higher-level cognitive processes (Goldenberg, 2013). In this perspective article, we will discuss just one of the possible pathological perspectives of the apraxic disturbance: whether the emerging concept of “agency” is consistent with the presentation of neurological symptoms due to apraxia. Distinct from other clinical disturbances such as anosognosia for hemiplegia—where the symptom of disownership (Karnath and Baier, 2010) with consequent disorders of motor awareness of the paralyzed parts have been interpreted in relation to agency (Pia et al., 2013)—the framework we offer here specifically involves a more global and genuine action volitional disorder that typically affects the two sides of the body. Until recently, a limited number of experimental studies have identified the essential aspects of agency that can be objectively quantified in apraxia by the following three lines of evidence: (i) a genuine incapacity with respect to the voluntary control of one’s action bound closer to its outcome; (ii) a disordered subjective experience of actions both performed and not; and (iii) altered predictive signals generated during motor planning.

The first evidence is related to the fundamental importance of performing an intentional action with an outcome and, secondly, to the subjective sense of control in the selection of actions. Consider, for example, the active action of taking a cigarette from a pack, opening a book of matches, and then lighting and drawing on the cigarette. A variety of movements may be similarly effective when it comes to performing the given aim of smoking. However, if an apraxic patient attempts to smoke, he typically exhibits poor control over actions, and has difficulty in movement selection, which is compatible with slow, incorrect, and ineffective motor acts (“put a match to the mouth in an attempt to smoke”, Pick, 1905). Given that the patient showed intact knowledge of functional uses of objects and a disturbance of the mental control of deliberate motor actions, Pick interpreted this disorder as a sign of apraxia. The patient generally recognizes that the action performed does not unfold as expected, and reports his disappointment. Phenomenologically, we can distinguish at least two aspects interpretable in relation to the sense of agency. The first aspect is a disorder of volitional movement where non volitional movement is spared. In the first description of apraxia, Jackson (1866) observed the core pathology as a motor purposeful deficit [“*the patient seems to have lost much of his power to do anything intentionally, even with those muscles that are not paralyzed”]*. The second aspect, related to the first, is the incapacity to select the correct action leads to a weak power over the outcome itself. Apraxic patients not only have problems with purposeful object manipulation in everyday activities, but also in selecting actions (Rumiati et al., 2001), demonstrating impairments not related to mere movement execution (Hermsdörfer, 2014), nor to a loss of functional semantic knowledge or resource limitation (Rumiati, 2014). In motor act selection, patients with apraxia lose much of their power to perform intentional actions, are more prone to errors and have typically prolonged response times compared to neurological controls without apraxia (Goldenberg and Hagmann, 1998; Goldenberg et al., 2004; Hermsdörfer et al., 2006; Rumiati, 2014). The fragility of the deliberate control of their own actions may substantially depend on the interference caused by the competition between varieties of degraded movements (Sirigu et al., 2004; Pazzaglia et al., 2008b; Botvinick et al., 2009; Buxbaum and Kalenine, 2010; Jax and Buxbaum, 2010; Nelissen et al., 2010). Thus, weakened movement representation impedes correct and fast action selection processing by reducing freedom and power in selecting between possible movement options, thereby contributing to a reduced fluency (Haggard and Chambon, 2012, for a review) and sense of agency over one’s own action effects (Wenke et al., 2010). Consistent with a deficit that implies a failure to select or retrieve stored internal representations, apraxia should affect the subjective perception of generated actions.

Another evidence is the disorder of self-generated action essential to establishing a sense of agency. A seminal paper showed experimentally that a sample of patients who had developed apraxic symptoms exhibited deficits in judging whether they did or did not cause a specific movement of their own body (Sirigu et al., 1999). With a more traditional experimental paradigm such as the explicit attribution in agency task, the patients were asked to execute simple and complex hand–finger movements with their unseen, gloved hand, and to observe in real time hand movements relayed on a video display. The display showed either the patient’s own hand or that of a model who performed the same movements. The apraxic patients were selectively impaired in deciding whether the hand moving on the screen was their own or belonged to someone else and become aware of their decision with a significant delay compared to healthy participants (Sirigu et al., 2004).

Different authors questioned the validity of these explicit judgments when studying agency, suggesting a more reliable, implicit quantitative measure for the awareness of action based on an intriguing relationship between voluntary action and subjective time (Haggard et al., 2002). This so-called “intentional binding” measure has been studied in patients with cortico-basal degeneration, some with clinical apraxia (Wolpe et al., 2014). Participants were asked to report either when they pressed a button or when they heard a tone. In the case of apraxic patients the intentional binding is associated with a subjective contraction of time between an action and its effect. This change in judgment is proportional to disease severity of apraxia but not to other motor features or cognitive impairments and occurs for the reduced sense of ownership of the action (Wolpe et al., 2014). Increased binding of action in patients is therefore more likely to reflect a deficit in control of actions by the anticipation of their effects. This possibility is explored by closer examination of action prediction in patients with apraxia.

The third evidence are disordered predictive signals, which are critical to the sense of agency (Blakemore et al., 2001). According to the “comparator” model, one makes a choice on the basis of a match between the predicted and actual sensory effect of one’s action (Chambon et al., 2013). It is possible that in previous studies, patients failed to compare between an internal model and the expected and actual sensory consequence of the action (Sirigu et al., 2004). Indeed, patients with apraxia are unable to mentally simulate movements of their own hands, (Sirigu et al., 1995) and in monitoring the early phases of movement planning (Sirigu et al., 2004), thus suggesting an impairment in anticipating the sensory consequences of manual movements. The readiness potential (RP), a marker of motor preparation that increases just before an observed movement (Kilner et al., 2003), was explored using electroencephalography in an elegant study on apraxic patients (Sirigu et al., 2004; Fontana et al., 2012). Apraxic patients passively viewed a series of short video clips showing a predictable hand moving on the basis of changes of colored objects. The results revealed a clear association between deficits typically present in patients with apraxia and the alteration to monitor the early planning phases of self-generated actions. Specifically, instead of showing the marker of motor preparation to self-generated movement observation-related events that was exhibited in control participants, no such RP was observed in patients with apraxia. Research has revealed that RP results from forward model predictions of the motor system precisely automatically preceding the movement’s onset (Kilner et al., 2003). Within this context, the lack of RP exhibited by patients indicates that the inability to predict the consequences of one’s own motor actions lead to inadequate online updating during actions (Pazzaglia, 2013a,b). The online information about movement is a prerequisite for the capacity to feel that one’s own body generates the event and has control over it and the discrepancy between the predicted and actual sensory feedback is directly associated to a distorted phenomenology in the experience of agency.

**Figure 1:**
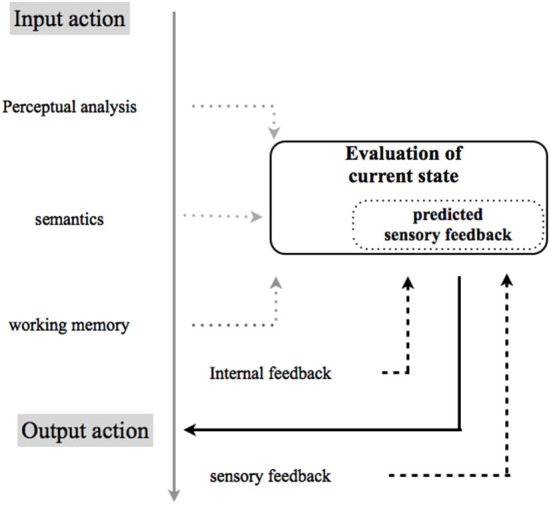
**Hypothetical model for performing and recognizing self-produced movements**. The model has been adapted from Rothi and Heilman (1997) within the internal model adapted from Sirigu et al. (2003). Failures in performing or in recognizing gestures may occur because of damage at any stage in the directional flow of action between perceiving (input) and performing (output) an action. Successful completion of any gesture-related task (e.g., execution, imitation or recognition of either correctly or incorrectly, transitive (using objects) or intransitive (without objects) meaningful conventional limb gestures, etc.) requires access to an internal model. A prominent theory in motor control proposes the use of internal models with capacity to control or predict the consequences of one’s own actions, and comparing these predictions to actual outcomes (Wolpert et al., 1995) adapted from Sirigu et al. (2003) to the putative level of dysfunction in apraxia. An efference copy of the motor command is used by forward models to predict the sensory feedback. The discrepancy between the predicted and actual sensory feedback is directly associated to a distorted phenomenology in the experience of agency. Hhypothetical models for performing and recognizing self-produced movements highlight the role of an internal model that attributes, evaluates, controls, or predicts the consequences of one’s own actions, and compares these predictions to actual outcomes.

Separately, the results from these studies reaffirm the objective difficulty in voluntary control of action and thereby its consequences by predictive mechanisms, and the ever-expanding apraxia picture on perturbation of agentive awareness. Despite few direct studies on agency, the disorder of the processes promoting agency that may co-occur in apraxia could fully explain the higher-order computations (e.g., related to intention to act and to the experience of controlling one’s own actions, and, through them, events in the outside world) that likely interact with low-level motor mechanisms (e.g., the automatic selection of action primitives on which conscious experience corresponding to efficiency of action selection is based). This hypothetical processing, necessary to account the different form of apraxia observed, may be predicted on the basis of an internal model (see Figure 1) that attributes, evaluates, controls, or predicts the consequences of one’s own actions, and compares these predictions to actual outcomes.

## Does agency play a crucial causative role in the left frontal–parietal network?

By examining both fMRI data on voluntary actions that are usually accompanied by an experience of agency and data on the anatomical localization of altered awareness and the control of volitional action in apraxia, it is possible to begin uncovering the neural substrates related to the sense of agency. FMRI allows the detection of brain activity changes that are *correlated* with motor intentions and subsequent action monitoring. It does not, however, clarify whether such activations play a *causal role*. In contrast, lesion-mapping analysis can highlight brain areas or circuits actively involved in the process of deriving actions from the original intention and plan of the movement. Several fMRI studies have suggested that the sense of agency, including action monitoring (Matsuzawa et al., 2005; Schnell et al., 2007; Farrer et al., 2008; Kontaris et al., 2009; Tsakiris et al., 2010; Chambon et al., 2013; Koban et al., 2013), prediction (Leube et al., 2003b; Ramnani and Miall, 2004; Spengler et al., 2009; Yomogida et al., 2010; Nahab et al., 2011), self-other coding (Blakemore et al., 1998; Leube et al., 2003a; Balslev et al., 2006; David et al., 2006, 2007; Ogawa and Inui, 2007; Fukushima et al., 2013; Lee and Reeve, 2013), and intentional binding (Kühn et al., 2013; Moore et al., 2013) involve the exchange of signals across a frontal–parietal network that voxel-based lesion symptom mapping (VLSM) analysis demonstrated is typically affected in apraxia (Pazzaglia et al., 2008a,b; Dovern et al., 2011). In particular, the posterior parietal cortex (PPC; Fink et al., 1999; Chaminade and Decety, 2002; Farrer and Frith, 2002; Farrer et al., 2003b, 2008; Chaminade et al., 2005) and the angular gyrus (AG) monitor signals related to action selection in the dorsolateral prefrontal cortex and the ventral premotor cortex (vPM) to prospectively signal subjective experience control over a coming action (Grossman et al., 2000; Leube et al., 2003a; Pelphrey et al., 2004; Ramnani and Miall, 2004; Saxe et al., 2004).

Healthy subjects report a decreased sense of agency when their intentions do not match the outcomes of their actions. In this case, activity in the temporo-parietal junction (TPJ) and AG regions increased as a function of the degree of retrospective action-intention mismatch (Farrer et al., 2008; Spengler et al., 2009), and might represent a self-indicator of volition prior to movement itself (Chambon et al., 2013). Therefore, direct electrical stimulation applied to the parietal cortex (AG and supramarginal gyrus, SMG) in patients undergoing awake surgery for tumor removal elicits the subjective experience of an “intention to move” the contralesional hand, arm, or foot (Desmurget et al., 2009).

Direct evidence of the anatomical and functional association on three different levels (the choice of action where ambiguity is present; self-perception/intentional binding; and the intention to act) has been obtained in patients with apraxia. Neurological investigations into the intention to move in apraxic participants have shown that the AG, in the inferior parietal lobule of the parietal cortex, may be essential for anticipating the multisensory consequences of predicted movements (Sirigu et al., 2004; Fontana et al., 2012). Similarly, both frontal and parietal structures may differentially code for self-generated actions as well as for action selection (Sirigu et al., 1999; Pazzaglia et al., 2008b). Patients with apraxia, who systematically identified the hand of a model that performed their same movement as their own, demonstrated mainly fronto-parietal lesions (Sirigu et al., 1999). A clear association was identified between the impairment in selection of four versions of actions to the gesture-sound with lesions mainly involving the inferior parietal region, SMG, and AG, but also extending as far as the frontal lobe (Pazzaglia et al., 2008b). Yet, impairments in correct selection of three versions of the same visual gesture presented within the same trial were related to the gray matter volume of the left anterior inferior parietal cortex extending into the posterior superior temporal gyrus (Nelissen et al., 2010).

**Figure 2:**
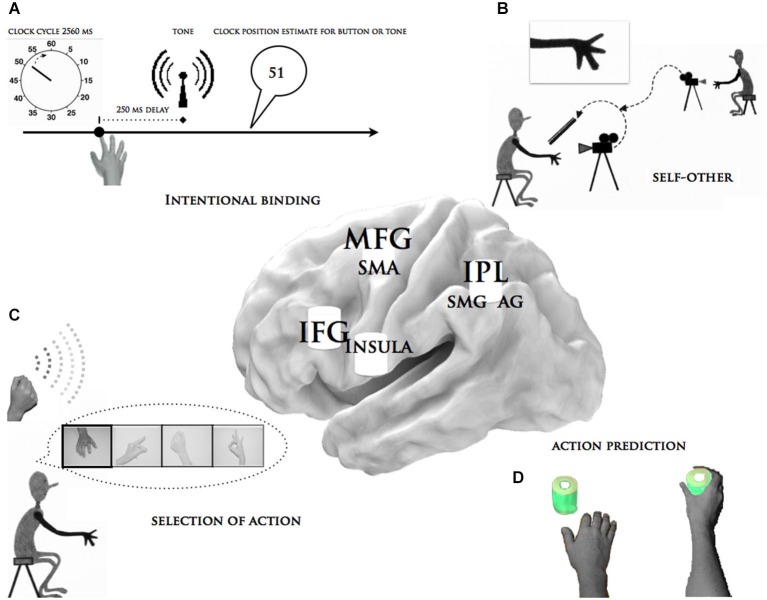
**Summary of the functional and anatomical link between the essential aspects of agency and apraxia**. This figure describes some of the objective measures of agency obtained in patients with apraxia: **(A)** The “*intentional binding*” paradigm, in particular the chronometric approach for volition. **(B)** The “*self vs. other*” paradigm, specifically the differentiation of self-generated movements from experimenter-generated actions. **(C)** The “*selection of action paradigm*”, particularly the feeling of less power over efficacy of one’s own action. **(D)** The “*neural intention to act*” in which EEG signals were recorded while the subjects passively watched a series of short videos showing the voluntary actions of an actor’s hand. The central brain illustration depicts the critical brain areas for apraxia, which are alluded to in these paradigms. MFG = middle frontal gyrus, SMA = supplementary motor area, IFG = inferior frontal gyrus, insula, IPL = inferior parietal lobe, SMG = supra marginal gyrus, AG = angular gyrus.

Another circuit, anchored in the frontal lobe involving the supplementary motor area (SMA) and its most anterior portion, the pre-SMA (Farrer and Frith, 2002; Farrer et al., 2003a,b; Haggard and Clark, 2003; Haggard and Whitford, 2004; Cunnington et al., 2006; Lau et al., 2006), together with the dorsolateral prefrontal cortex (Fink et al., 1999; Slachevsky et al., 2001; Schnell et al., 2007; Synofzik et al., 2008), has been proposed to play a role in intentional binding and the judgment of agency, as has the insula (Karnath and Baier, 2010). Only recent behavioral, structural, and functional results converge to reveal the frontal network for altered awareness and the control of voluntary actions in patients with apraxia (Wolpe et al., 2014). Structural neuroimaging of voxel-based morphometry and diffusion tensor imaging showed that the volitional signals that drive internally generated actions in an intentional binding task were modulated by gray and white matter degeneration in the medial frontal-prefrontal network, with its hub in the pre-SMA (Wolpe et al., 2014). In apraxic patients, the dorsal premotor cortex may be essential for intentionally retrieving motor knowledge (Dovern et al., 2011). Although a substantial proportion of right-hemisphere damaged patients also showed apraxia (Donkervoort et al., 2000), involvement of the right hemisphere lesions to the sense of agency is currently lacking. Thus, two regions in the left hemisphere process different information (Figure 2), while the parietal lobe’s principal functions might be to self-monitor motor intentions, the frontal lobe might be directly involved in forming, monitoring, and control intentions. Nonetheless, other cortical areas, such as the insula (Pazzaglia et al., 2008a,b), have been implicated in selecting different actions, so that the agentic experience in apraxia is likely to be sustained by a distributed left brain network rather than by a single brain center. For a schematic representation of the functional and anatomical link between the essential aspects of agency and apraxia see Figure 2.

## Automatic retrieval strategies in therapy for apraxia rehabilitation

The loss of a sense of control over one’s own movements plays, in apraxic patients, an important role in many purposeful actions that are an inherent part of daily life. It affects the self-care routines (Foundas et al., 1995; Hanna-Pladdy et al., 2003; Walker et al., 2004; Smania et al., 2006) with respect to, for example, personal hygiene (Goldenberg and Hagmann, 1998), preparing food (van Heugten et al., 2006), eating (Foundas et al., 1995), and dressing (Walker et al., 2004). Occasionally, the inability to predict the consequences of one’s own motor acts can have devastating effects that jeopardize the autonomy and safety of the individual (Giovannetti et al., 2002; Hanna-Pladdy et al., 2003; Bettcher et al., 2011). A person with apraxia might be able to safely eat candy, but when attempting to smoke, risks getting burnt on the palm of the hand, cheeks, or elsewhere. From an adaptive point of view, intentional selection about incorrect actions could be deeply pervasive in a patient’s life, and sometimes dangerous for their own safety (Hanna-Pladdy et al., 2003).

It is clear that progress in understanding action awareness and control represents a significant opportunity to strengthen the automatic rather than intentional retrieval strategies in the treatment of apraxic patients. After a stroke, patients with apraxia must increase the capacity to automatically retrieve learned motor knowledge by restoring the congruency between sensory-motor and intention systems. The prospective sense of agency might only develop once the brain has automatically re-learned. Matching or mismatching between visual but also multimodal signals and motor output re-stabilizes the relation between actions and outcomes. Automatically re-learning the appropriate responses to familiar action situations using closely associated perceptual-motor codes permits patients with apraxia to improve their selection of action (Smania et al., 2006), and thus function independently, but also, more importantly, can block the generation of unsafe motor patterns (Hanna-Pladdy et al., 2003).

## Concluding remarks

Taken together, the studies discussed in our perspective article seem to reveal a picture of apraxia that, although probably still incomplete, demonstrates how altered mechanisms that underlie awareness and control can be detrimental to agency. As such, these studies disclose more about agency itself. The prospective framework we offer here for apraxia renew the interpretation of the puzzling aspect generally viewed as apraxia, and encourage the advancement of novel and effective treatments to cure the disorder. Moreover, for agency, we provided support for the existence of a left parietal–frontal network underlying agentive self-awareness that continues to be a valuable way for gathering conclusive evidence on the role of agency in motor control and cognition as a natural part of human life, and thus provide ecologically valid data. Future studies focusing specifically on the thematic content of the sense of agency (e.g., related to the control of one’s own action or to intention to act to social and cultural conditions in which the idea of responsibility is central for our own actions) may help to understand the wide and complex range of human actions in both normal and pathological conditions.

## Funding information

This work was supported by the University of Rome “Sapienza” and the Italian Ministry of Health [grant RC13.G] by IRCCS Fondazione Santa Lucia.

## Conflict of interest statement

The authors declare that the research was conducted in the absence of any commercial or financial relationships that could be construed as a potential conflict of interest.
